# Supplementary material for: Are carbon emissions trading and green financial instruments synergistic? -Comprehensive quantitative research based on content analysis
Source: PLoS One. 2024 Mar 7;19(3):e0298601. doi: 10.1371/journal.pone.0298601 (PMC10919601; doi:10.1371/journal.pone.0298601)
Supplement: S1 Data — (ZIP) [file pone.0298601.s001.zip › Data/地级市Z_F工作报告.zip!/║╙─╧/╞╜╢Ñ╔╜╩╨/└┤╫╘╣1⁄2╓┌║┼í╛┬φ┐╦╩2╛▌═°í┐_2003.docx]

各位代表：

现在，我代表市人民政府，向大会作工作报告，请予审议，并请各位政协委员及其他列席人员提出意见。

一、２００２年工作回顾

２００２年，我们在省委、省政府和市委的正确领导下，带领全市人民高举邓小平理论伟大旗帜，全面贯彻“三个代表”重要思想，围绕“建设大城市，发展大工业”的目标，团结奋斗，扎实工作，较好地完成了市七届人大四次会议确定的各项任务，国民经济继续保持良好的发展势头。全年预计完成国内生产总值３２１．１亿元，增长８．７％。其中，第一产业增加值４５．８亿元，增长３．９％；第二产业１７１．１亿元，增长１０．７％；第三产业１０４．２亿元，增长７．８％。

农业结构调整取得新进展，农村经济持续发展。预计完成农林牧渔业总产值７８．１４亿元，增长４．１％；粮食总产量１６２．９６万吨。农业结构调整进展顺利，种植业效益明显提高，优质高效粮经作物面积扩大。农业生产条件继续改善，新增有效灌溉面积５．２５万亩、旱涝保收田５．１万亩新建水窖及小型水源工程３０４０处解决了１２万人的饮水困难。大力开展植树造林完成造林面积２５．２万亩其中退耕还林１７．８万亩。畜牧饲养业有较大发展肉、蛋、奶类总产量分别增长９．９５％、８．７％和２４．２％。乡镇企业保持良好发展态势，预计完成增加值８９亿元，增长１０．７％。农村税费改革进展顺利，农民总体减负３８％。

工业经济快速增长，企业改革进一步深化。预计完成工业增加值１５７．１亿元，增长１０．７％。其中，限额以上工业企业完成增加值８５．６亿元，增长１４．５％；产销率为９９．３％。原煤、焦炭、帘子布、水泥、钢材等产品产量增长较快。经济效益进一步改善，限额以上工业企业实现利税１８．６亿元，增长２７％；其中利润４．２亿元，增长８８．９％。宝酒集团与健力宝集团成功合作。平煤、飞行、星峰集团债转股工作已经完成。神马集团债转股工作取得重大进展。中小企业改革步伐加快，市直２０户企业落实了改革方案，其中１０户企业实施了破产，市被单厂、印刷厂、非织造布总厂、韩庄矿务局破产终结并进行了资产重组。县（市、区）中小企业改制面达９３．８％。

固定资产投资规模扩大，重点项目建设进展顺利。预计完成全社会固定资产投资６０．３亿元，增长１０．１％。姚电公司１号机组改造、三和热电公司２号机组、天瑞集团年产１００万吨干法水泥生产线、汇源公司氢氧化铝二期、汝州市污水处理厂、城市电网改造、昭平台灌区配套三期、湛南国家粮食储备库等工程已经建成；平煤集团田庄选煤厂技改、农村电网改造二期、田岗水库复建等项目即将完工；许平南高速公路境内段、舞钢公司西气东输管线钢材技改、天鹰集团超高压输变电设备、叶县蓝光电厂一期、白龟山和昭平台水库除险加固等项目完成了年度投资计划。新建、改建国家和省干线公路２５３公里，县道二级路５２３公里；“村村通”工程完成四级油路１４６０公里，解决了６３０个行政村通油路问题，汝州、舞钢、宝丰实现了平原地区村村通油路。

城市建设力度加大，城市面貌有新的改善。城建“八大工程”进展顺利，建设路创建迎宾大道东段已经通车，新城区７．６平方公里控制性详细规划通过评审，快速通道、湖滨大道、高阳路等工程正在加紧施工。积极拓宽融资渠道，与中原信托投资公司达成共建新区融资平台协议。劳动路、湛南路退路进场和老百货楼商业区改造进展顺利，完成了凌云路、湛北路西段、鹰城广场西路等道路的新建改造，以及湛河公园、山顶公园、鹰城广场、河滨广场、焦庄垃圾填埋场等续建工程。实施“蓝天工程”，市区空气质量有所改善。加快城市管网改造建设，改造供水、供热、燃气管网２０．７公里。开展了市容市貌综合整治，城市面貌有新的改观。

第三产业发展步伐加快，对外开放取得新成绩。预计完成社会消费品零售总额１０２．５亿元，增长１０％。流通现代化发展迅速，商业贸易、交通运输、邮电通信等行业发展势头良好。平顶山贸易广场、花卉大世界等市场建成投入使用。旅游业总收入增长１３．６％。石人山景区晋升为国家重点风景名胜区，石漫滩水库和昭平湖景区被评定为国家水利风景名胜区。完成外贸进出口总值１．５１亿美元，增长２１．５％，其中出口８６１３万美元，增长９０．１％。招商引资力度加大，成功举办了香港“平顶山市对外经济技术合作项目洽谈会”。全年合同利用外资２０６６万美元，增长３４．１％；实际利用外资１８８３万美元，增长３６．４％。

财政金融运行平稳，社会保障工作得到加强。完成地方财政收入１２．３８亿元，按可比口径计算，增长８．１％；财政支出２４．６１亿元，增长２４．１％。金融机构年末各项存款余额２５９．４８亿元，比年初增长１９．４％；贷款余额１９２．７７亿元，增长１１．４％。大力实施再就业工程，全年开发就业岗位１６７２１个，安置下岗失业人员９７９１人。严格落实“两个确保”，全年发放“两金”４．３２亿元，按时足额发放率和社会化发放率均达１００％；发放城市“低保”资金３３０２万元，实现了应保尽保。城镇居民人均可支配收入６０８０元，增长１５．３７％；农民人均现金收入２２０９．８２元，增长５．２２％。

整顿和规范市场经济秩序成效明显，安全生产形势稳定好转。以打假打诈和企业经营环境整治为重点，捣毁了一批制售假冒伪劣商品的窝点，集中查处了一批“四乱”案件。对加油站和集贸市场进行了重点整治。进一步加大了煤矿安全生产管理力度，严防死守责任制得到较好落实，煤矿安全生产形势进一步好转。非煤矿山、公共聚集场所、消防、民爆物品和烟花爆竹、道路和水上交通、危险化学品储运等方面专项治理成果明显。全年事故起数和死亡人数分别下降８．３％和４５．９％，其中煤矿事故和死亡人数分别下降１６．７％和６３％，受到省政府和国家有关部委的表彰。

各项社会事业全面进步，精神文明和民主法制建设进一步加强。继续实施“科教兴平”战略，加大科技创新投入，获得市级以上科技进步成果奖１９９项。全面推进素质教育，基础教育改革迈出新步伐，教育教学质量有新的提高，高等教育取得新成绩，平顶山工学院挂牌成立。文化、卫生、体育、广播电视事业健康发展。成功地举办了汝瓷邮票首发式。承办了河南省全民健身宣传周开幕式暨体育进社区演示大会。市广电中心建成投入使用。医疗质量和服务水平不断提高，连续四年荣获省医疗质量管理先进市称号。舞钢、宝丰分别被评为省级卫生城市和卫生县城。计划生育综合改革试点工作进展顺利，人口自然增长率下降到６．３‰，连续八年被评为省计划生育工作先进市。“双拥”和军民共建活动进一步深化，我市及汝州、鲁山、宝丰、舞钢分别被省政府评为双拥模范城。

深入开展形式多样的群众性精神文明创建活动，城乡文明程度和公民素质逐步提高。积极实施“四五”普法和“二五”依法治市规划。第四届村委会换届选举工作基本完成。社区建设取得显著成绩，卫东区被命名为全国社区建设示范区。机构改革稳步推进，人事制度改革进一步深化。全面推行政务公开，市行政审批服务中心挂牌运行。认真执行人大及其常委会的决议、决定，自觉接受人大、政协监督，全年办理人大代表建议、政协委员提案４００件。重视人民群众来信来访，完善了市长热线电话网络。强化社会治安综合治理，保持了社会大局稳定。民族宗教、统计、审计、外事、侨务、档案、对台、人防、民兵、新闻出版、史志、气象、物价、盐业、国家安全等项工作都取得了新的成绩。

２００２年经济和社会发展的显著成绩，确实来之不易，这是省委、省政府和市委正确领导的结果，是市人大、市政协监督和支持的结果，是全市人民锐意进取、团结奋斗的结果。我代表市人民政府，向辛勤工作在全市各条战线上的广大工人、农民、知识分子、干部、人民解放军、武警官兵和公安干警以及关心、支持平顶山建设的各界人士，致以崇高的敬意和衷心的感谢！

我们也清醒地看到，当前经济和社会生活中还存在不少亟待解决的问题。主要是：农民和城镇部分居民收入增长缓慢，部分企业生产和职工生活仍然困难，就业压力大。经济结构性矛盾仍然存在。财政收支矛盾突出，县乡财政还十分困难。清理整顿“三会一部”及企业不规范集资工作中的困难和问题还比较多。城市建设和管理方面存在的问题仍然非常突出。有些地方社会治安状况不好。少数领导干部的形式主义、官僚主义尤其是工作不落实问题和弄虚作假、铺张浪费行为相当严重，极少数干部甚至贪污腐化。这些问题，有的是长期积累下来的，有的是体制转轨和结构调整过程中难以完全避免的，有的是由于工作中的缺点和不足造成的。今后要继续采取措施，努力加以解决。

二、２００３年政府工作的总体要求和主要奋斗目标

今年政府工作的总体要求是，以邓小平理论和“三个代表”重要思想为指导，认真贯彻党的十六大精神，紧紧围绕全面建设小康社会的战略目标，按照“建设大城市，发展大工业”的要求，继续打好六大攻坚战，做好十项重点工作，进一步扩大内需，调整结构，深化改革，加快开放，优化环境，大力推进新型工业化，全面加快城镇化进程，促进农村经济全面发展，推动国民经济持续快速健康发展，实现较高的增长速度和较高的增长质量。正确处理改革发展稳定的关系，切实做好就业再就业和社会保障工作，提高城乡居民生活水平。继续实施科教兴平、开放带动和可持续发展战略，大力加强社会主义精神文明和政治文明建设，促进社会全面进步。

今年全市经济社会发展的主要奋斗目标是：国内生产总值增长９％，全社会固定资产投资增长１０％，地方财政收入增长７％，社会消费品零售总额增长１０％，外贸出口总值达到８０００万美元，城镇居民人均可支配收入增长８％，农民人均纯收入增长５％，居民消费价格总水平上涨１％左右，城镇登记失业率低于４％，人口自然增长率控制在７‰以内。

从现在到２０２０年，要实现党的十六大提出的全面建设小康社会的奋斗目标，必须坚持以经济建设为中心，把发展作为第一要务。在优化结构和提高效益的基础上，确保我市国内生产总值比２０００年翻两番以上，力争人均国内生产总值接近或达到全国平均水平，非农劳动力占劳动力总数的比重和城镇人口占总人口的比重分别达到６０％和５０％以上，基本实现工业化和城镇化。

要完成上述目标，我们面临的困难和问题还比较多，任务十分艰巨。因此，今年和今后一个时期政府工作要始终把握好以下几个事关全局的重大问题。

第一，紧紧围绕发展大工业，把培育八大工业基地作为实施工业强市战略的重大举措，突出大项目建设，深化国有企业改革，大力推进新型工业化，把优势企业做大做强。

第二，紧紧围绕建设大城市，加大经营城市力度，在新城区建设、老城区改造和城市管理及小城镇建设上取得突破性进展。

第三，紧紧围绕农民增收和农业现代化，把县域经济发展摆到更加突出的位置，大力推进农业和农村经济结构调整，突出比较优势，培育主导产业，做大做强龙头企业，逐步形成项目带动、分区推进的特色农业格局。

第四，紧紧围绕增加经济总量和就业容量，毫不动摇地鼓励、引导、支持非公有制经济和第三产业实现跨越式发展，使之真正成为新的经济增长点和扩大就业的主渠道。

第五，紧紧围绕扩大开放和优化发展环境，全面实施“引进来”和“走出去”战略，加大招商引资力度，加强服务型政府和企业发展环境建设，树立平顶山对外开放的新形象。

三、加快经济结构的战略性调整，促进国民经济持续快速健康发展

（一）走新型工业化道路，提高工业经济增长的质量和效益

走新型工业化道路是提高工业经济增长质量和效益的必然选择。今年，我们要在探索新型工业化的实现途径上取得重大进展，强力推进八大工业基地建设。

第一，要以改造提升八大支柱产业为切入点，加快用高新技术和先进适用技术改造传统产业步伐，重点发展化工及机电装备业产品，使其成为我市战略主导产业。以高附加值关键项目为依托，加速资源优势与生产要素的紧密结合，巩固提升煤炭、电力、纺织、冶金、建材、食品等产业，走集约化、规模化发展道路，充分发挥比较优势，促进工业结构的优化升级。

第二，要抓住“改造提升”的结合点，大力开展企业技术创新工作。各企业要树立强烈的机遇意识和竞争意识，找准市场定位，坚持“质量、品种、效益”和“替代进口、扩大出口”的主攻方向，切实加大技改投入。对已确定的２７个重点技改项目，要采取“项目定人、专人分包”的办法强力推进，切实发挥技术改造的倍增效益。

第三，突出重点，有选择地发展高新技术产业。继续将高新技术及产业化工作重点向八大工业基地倾斜，对平煤、神马、舞钢、天鹰、电收尘器厂等企业在高新技术领域开发出的战略性产品，要集中力量重点扶持，加速产业化步伐。对进行应用技术开发的民营科技企业，如康立电气公司、舞钢联能系统有限公司等，要切实加大扶持和开发力度。发挥信息技术的先导作用，加速信息技术与企业科技进步的结合。对具有潜在优势的新材料及环保领域的高新技术产品，如煤矸石、粉煤灰综合利用等项目，要积极为其规模生产创造条件，培育后发优势。

第四，大力实施名牌战略。充分发挥政策性资金的导向作用，积极争取省工业结构调整专项资金、高新技术产业化资金和中小企业创业基金，对省市级名牌产品进行持续扶持，发挥优势产品的潜能，通过产品延伸、产品扩散、技术转让、协作配套等形式，尽快形成一批市场占有率高、生产规模大的名牌产品。

大项目建设是支撑我市经济增长的关键所在。要紧紧围绕培育八大工业基地，着力抓好一批工业结构调整重点工程。平煤集团３０万立方米粉煤灰混凝土砌块、舞钢公司西气东输管线钢材技改、天鹰集团超高压输变电设备和轨道交通电器产品、中加钢铁公司５０万吨炼铁、叶县蓝光电厂一期、安钢舞阳矿业公司赵案庄矿二期等７个项目，要确保年内建成投产。平煤集团洗配煤中心、神马集团１２．５万吨尼龙６６盐扩建与中间产品开发和６０００吨涤纶工业丝、鸿翔公司２×２０万千瓦热电机组、星峰集团年产１００万吨熟料水泥窑外分解生产线、天使集团莫代尔高档竹节服装面料生产线等６个项目，要尽快开工建设。姚电公司２×６０万千瓦发电机组、神马集团５万吨离子膜烧碱及ＰＶＣ专用树脂改造、平煤集团八矿二号井、电收尘器厂脱硫除尘设备国产化、天瑞集团年产２００万吨新型干法水泥生产线和２×１３．５万千瓦发电机组及铁路重载高速机车重要配件、天使集团年产２０００万米高档色织服装面料生产线、叶县蓝光电厂二期、平煤集团坑口电厂二期、汇源公司氧化铝等１１个项目，要千方百计加快前期工作进度。

（二）积极推进农业和农村经济结构调整，全面振兴农村经济

推进农业和农村经济结构调整是新阶段农村经济发展的中心任务。今年，我们要在培育支柱产业、壮大龙头企业和发展高效农业上取得新的突破，形成特色突出、布局合理、服务健全的农业产业化新格局。

一是要强力发展畜牧业、林果业，在培育支柱产业上取得突破。紧紧围绕把我市建设成为全省重要的畜产品生产和加工基地的目标，突出牛羊猪禽四大产业，建立和完善良种繁育、疫病防治、畜产品加工等服务体系，重点抓好沙汝河肉牛肉羊育肥带、舞钢肉鸡产业化开发、外销生猪产业化、近郊奶业、５０万亩秸秆牧草等八大工程建设。全市新增大型规模饲养企业１６户，饲养园区６５个，示范乡镇２５个。每个县（市、区）都要发挥比较优势，着力培育一个主导产业，力争通过２到３年的努力，使叶县肉牛、舞钢肉鸡、宝丰生猪、郏县红牛、汝州蛋鸡、鲁山山绵羊、近郊奶业初步形成产业化基地生产格局，使畜牧业真正成为振兴县域经济的突破口。当前，林业建设出现了前所未有的发展机遇。要进一步明确林权，落实政策，以退耕还林为重点，加快荒山造林、平原绿化、城郊绿化、通道绿化步伐，完成２２万亩退耕还林任务，争取在较短的时间内，使我市的森林覆盖率、林木蓄积量和林果业产值有较大增长。

二是要积极发展现代农业、特色农业和观光农业，在发展高效农业上取得突破。城市近郊重点扩大蔬菜、名优水果和苗木花卉种植规模，三年内经济作物面积要达到８０％以上。沿国道、省道两侧发展蔬菜、苗木、花卉，形成绿色走廊。沙汝河平原区重点发展优质高产高效小麦、玉米、油料、烟叶等作物，全市优质专用小麦和玉米面积要分别稳定在１００万亩，油料作物面积要达到６０万亩。丘陵易旱地区重点发展耐旱作物，优质小杂粮基地面积要达到７０万亩。每个县（市、区）要建立１至３个高科技农业示范园区。通过优化区域布局，建立优质小麦和玉米、优质小杂粮、优质油菜、优质蔬菜、优质瓜果、优质烟叶、食用菌、水产品、各类花卉、观光农业十大生产基地，加快形成具有我市特色的农业园区和产业带。

三是大力推进农业产业化经营，在培育龙头企业上取得突破。适当增加并集中使用农业结构调整专项资金，重点支持优质农产品、畜产品生产基地建设和加工龙头企业发展。落实优惠政策，扶持市豫美面粉有限公司、舞钢华懋双汇公司、万东牧业有限公司、叶县肉牛系列加工企业、宝丰康龙公司、汝州巨龙淀粉厂等一批规模大、带动能力强的农产品加工贸易企业，形成一批具有竞争力的龙头企业。每个县（市、区）都要扶持３至５个重点龙头企业。大力发展农业专业合作经济组织和农民经纪人队伍，加快区域农产品市场和专业市场建设，建立农产品检验检测体系和质量标准体系，提高农业专业化水平和农民进入市场的组织化程度。加快乡镇企业改组改造和技术进步，引导乡镇企业向县城和小城镇集中，促进城乡一体化，壮大县域经济。

坚持党在农村的基本政策，依法稳定农村土地承包关系。按照依法、自愿、有偿的原则进行土地承包经营权流转，逐步发展规模经营。巩固和深化农村税费改革，确保农民负担不反弹。继续推进粮食流通体制改革，切实保护农民利益。做好扶贫开发和以工代赈工作，努力实现贫困人口脱贫。继续抓好以水利为重点的基础设施建设，重点搞好农村饮水工程和白龟山、昭平台、老虎洞水库除险加固及昭平台、孤石滩灌区节水灌溉配套、郏县广阔渠复建等水利工程建设。配合国家建设南水北调工程，做好燕山水库的前期准备工作。全年新打配套机井３０００眼，新增有效灌溉面积５万亩、旱涝保收田６万亩，发展节水灌溉面积６万亩，治理水土流失面积９０平方公里，解决１１．７万饮水困难群众的饮水问题。

（三）努力扩大投资和消费需求，大力发展第三产业

加快重点项目建设，靠项目加快发展，靠项目优化结构，靠项目增强发展后劲。严格落实重点项目建设责任制，对今年确定的５８个重点项目，要集中力量，强力推进。第四水厂、城市垃圾无害化处理工程要确保上半年投入运行。年底前，要确保农村电网改造二期、平桐线改建等项目建成投入使用。许平南高速公路境内段、平顶山工学院和师专扩建等项目要完成年度投资计划。漯平、平洛高速公路和西气东输天然气利用、平东车站改扩建、鲁山县城至石人山道路改建、市第一人民医院病房大楼等项目，要确保年内按期开工。对正在进行前期工作的项目，要实行全程服务，加快工作进度。加强项目库建设，论证、储备一批事关我市经济结构调整、产业升级的项目，为长远发展备足后劲。积极争取上级对公路建设的优惠政策，继续打好交通建设攻坚战。全年完成国家和省干线公路２０５公里；新修改建县道二级公路４３０公里。修建“村村通”公路７３８公里，解决３２６个行政村不通油路问题，除少数偏远山区外，实现村村通油路。

彻底清理和消除限制投资与消费的各种障碍，完善鼓励和引导投资与消费的政策措施。鼓励扩大住房、信息通信、旅游休闲、教育培训、社区服务、体育健身和卫生保健等领域的消费。推行购房入户政策，全面放开住房二级市场，积极培育房屋租赁市场。建立多元化的投资体制，吸引更多的社会投资主体参与交通、水利、基础设施的建设和经营。凡是国家法律、法规没有明令禁止的行业，都要向民间资本开放。

以旅游业为突破口，带动第三产业全面发展。要把做大做强旅游业作为经济结构调整的重要举措，充分发挥我市旅游资源丰富的优势，认真制定旅游业发展规划，高水平搞好景区建设，力争石人山通过国家４Ａ级景区验收，石漫滩、昭平湖、叶县明代县衙、三苏园等景区通过２Ａ级验收，加快风穴寺、香山寺等景区（点）的建设进度。加强宣传推介，强化旅游促销，争取用２至３年的时间，把我市建成国家优秀旅游城市，舞钢、鲁山等建成优秀旅游强县（市）。积极推进电子商务、通信、金融、保险、会计、法律、咨询、科技服务、房地产、家庭装饰等现代服务业，加快发展教育、文化、卫生、保健和体育事业。继续发展交通运输、仓储批发和零售贸易、餐饮、修理等传统服务业，大力发展家政服务、托老托幼、生活护理、社区保安、保洁保绿等社区服务业。加大商贸企业改制力度，促使国有商贸企业转机建制、退出国有。引导组织大中型零售企业，不断发展连锁经营、物流配送等新的营销方式，实施特色经营，努力提高经济效益和竞争实力。

（四）大力实施城镇化战略，不断提高城市经营管理水平

按照建设大城市的要求，高起点搞好城市规划。城市规划，特别是新城区规划要体现平顶山新兴工业城市的形象，必须组织国家级专家进行论证、设计，并广泛征求社会各方面意见，经审查批准后实施。抓紧做好城市总体规划的修编工作，完成新城区近期建设规划、城市景观规划和起步区详细规划。加大规划执法力度，坚决制止违规违法建设行为。

今年城市建设的重点是发展新城区和继续抓好“八大重点工程”。要全力抓好建设路创建迎宾大道工程，坚决拆除影响工程建设的建筑物，做好沿线建筑物的彩化、美化、亮化以及其他附属工程建设，确保完成高阳路东段、湛河治理三期１．２公里的建设任务，建成城东河、开发路、公园北街等三座大桥，使高阳路两侧１０多平方公里的区域得到开发利用。完成湛南路、劳动路等道路的“退路进场”及老百货楼商业区改造建设，组织实施对联盟路周围进行成片开发。加快湖滨大道、新城区干道及快速通道、西部生态园建设，基本完成新城区“七纵七横”道路建设和基础设施建设，尽快实现“五通一平”；市政大厦、公务员小区、新区广场等重点工程要力争尽快开工，推进新城区景观与环境建设，使新城区的起步区早日形成规模。同时，认真做好山顶公园、鹰城广场、河滨广场等项目的配套、绿化工程及老城区主次干道的修复维护工作。由平煤集团组织实施，完成光明路延伸至程平路工程。继续搞好水、电、气、热、通信等基础设施和公用设施建设。

树立经营城市的理念，多渠道筹措城市建设资金。继续完善土地储备制度，加强土地资产管理，控制好土地一级市场，对经营性用地实行统一规划、统一征地、统一出让的土地供应办法，用于开发经营的土地一律进入市场实行招标拍卖、公开交易。加大招商引资力度，对城市基础设施实行有偿使用，对道路、桥梁、街道、广场冠名权、广告权实行有偿转让，力争更多的外资、社会资金参与城市建设和市政公用设施建设。

下大决心抓好城市管理。要以创建全国卫生城市和省级文明城市为目标，搞好市场规划和建设，坚决清理“占道经营”，取缔“马路市场”；加大整治力度，解决“垃圾围城”和“乱搭乱建”，坚决改变脏乱差面貌。要排除一切干扰，坚定不移地推进城市管理体制改革，必须明确市、区、街及有关职能部门管理城市的责任和权利，把社区管理、“门前三包”纳入目标管理，严格奖惩措施。将垃圾收集、运输、处理和保洁、园林绿化及城市维护工程逐步推向社会，形成城市长效管理机制。引入竞争机制，加快公用事业市场化进程；优化公交线路，整顿市区客运市场。

进一步加快城镇化进程。按照优先发展中心城市，重点发展县级市、县城，选择发展建制镇的要求，今年我们要突出县级市、县城和重点镇建设，有选择地搞好小城镇规划，合理使用财力，引导乡镇企业、各类专业市场向县城和小城镇集中，形成产业支撑，带动和繁荣周边农村经济。

（五）进一步解放思想，强力推进非公有制经济发展

非公有制经济是我市经济发展的希望所在。要毫不动摇地鼓励、引导和支持非公有制经济的健康发展。积极鼓励非公有制企业参与国有经济结构调整、国有企业改革和农业产业化经营，发展龙头企业和中介服务组织。鼓励非公有制经济在从事传统第三产业的同时，积极向物流配送、旅游开发、信息咨询等现代服务业拓展。有序引导非公有制经济投资支柱产业、基础产业等领域，参与能源、交通、城市公共设施建设。对有一定规模和发展前景的非公有制企业，在制定规划、选择项目等方面分类指导，帮助解决资金融通、资产重组、人才流动等方面的问题。积极支持天瑞集团、汇源公司、华利药业公司、银龙集团、舒莱卫生用品公司、郏县城关苗木基地、洁石地板砖厂、刘家皮鞋厂等民营企业，通过新上项目和低成本扩张把企业做大做强。引导具有一定规模的私营企业加大技改投入，加强企业管理，大胆进行股份制改革，向现代企业制度迈进。支持民营科技企业、出口创汇企业实现规模生产，加快发展科技型、外向型企业。着力培育一批年销售收入超亿元的大企业和企业集团，实现非公有制经济增加值在国内生产总值中的比重大幅度提高。加大政策扶持和依法保护力度，使非公有制经济在投融资土地使用、经营环境等方面真正实现与公有制经济公平竞争，为非公有制经济发展创造宽松环境。

（六）深化国有企业改革，加快机制和管理创新

按照国家统一部署，稳步推进国有资产管理体制改革。继续实施大公司、大集团战略，通过市场和政策引导，全面提高平煤、神马、姚电、舞钢、天鹰等具有发展潜力的大型企业的核心竞争力，提高其在国内外市场上的知名度。已进入全省综合实力１００强的企业，要确保位次前移；没有进入的要加快发展，争取进入。按照建立现代企业制度的要求，加快国有企业规范的股份制改造，完善法人治理结构，做好债转股企业的转机建制工作。大力支持民营资本、社会资本和国外资本参与国有企业改组改造，形成多元化的持股结构，发展混合所有制经济。积极推进企业的体制、技术和管理创新，进一步提高企业管理水平。

要坚定不移地抓住产权制度改革这个关键环节，以依法破产和股份制改造为重点，加快中小企业改革。对于破产、兼并、收购、租赁、承包及发展股份合作制等形式的企业改革，都要按照“三个有利于”的标准，敢于打破旧的条条框框，勇于实践，大胆突破。对长期亏损、资不抵债、扭亏无望以及资源枯竭、无法转产的企业，坚决依法实施破产。

（七）坚持“引进来”和“走出去”相结合，全面对外开放

进一步扩大利用外资规模，提高利用外资质量。继续改善投资环境，创新利用外资方式，在交通、电力、城市公用设施、旅游业等领域运用项目融资、经营权转让、企业购并等方式鼓励外商投资。选择有条件的优势企业和项目，公开对外资特别是大型跨国公司招标转让股份。努力吸引境内外企业投资农业、制造业、高新技术产业和服务业。按照“政府牵头、企业自愿”的原则，全力以赴开展招商引资，精心组织好到国外、境外及国内发达地区的招商引资活动。充分利用我市丰富的资源和有利条件，吸引东部地区人才、技术和资金，加快我市资源的开发利用，改造传统产业，促进产业升级。进一步调整优化出口商品结构，在保持和发展帘子布、钢板、焦炭等出口商品份额的同时，抓好高压电器、电收尘器产品的出口，努力扩大农产品和其他产品出口。鼓励有竞争优势的企业到境外承揽工程、开展劳务合作。

（八）努力做好增收节支工作，服务经济发展

严格依法治税，强化税收征管稽查。建立财源监控网络，加强对重点行业、重点税源的监控，加大对第三产业税收的征收力度。严格对非税收入的管理，规范归集财政性资金。大力清理欠税、严厉打击偷税、逃税、骗税、抗税等违法犯罪行为。深化“收支两条线”管理，行政事业单位的行政性收费收入要纳入预算，实行收支脱钩管理。继续调整财政支出结构，大力压缩一般性支出，确保行政事业单位人员工资发放，加大对社会保障、教育、科技、农业、基础设施建设等公共需要的保障力度。办好市国库集中收付中心。认真贯彻执行《政府采购法》，进一步完善政府采购制度。

充分发挥金融对经济发展的支持作用。引导金融机构不断优化贷款结构，提高金融服务质量，加大对企业改革、农村经济、城市建设、第三产业的支持力度。完善贷款担保机制，解决中小企业和民营企业贷款难问题。推进金融安全区建设，帮助和支持金融机构进一步降低不良贷款比例，防范和化解金融风险。

（九）千方百计扩大就业，健全社会保障体系

各级政府必须把改善创业环境和增加就业岗位作为重要职责，坚决落实税费减免、贴息贷款、社保补贴、就业援助等下岗失业人员再就业扶持政策。鼓励发展就业容量大的个体、私营、外商投资、股份合作等多种所有制经济，发展有市场需求的中小企业和劳动密集型企业，鼓励国有大中型企业实施主辅分离和辅业转制，强力开发社区就业岗位，扩大就业总量。全年开发就业岗位４万个。引导全社会转变就业观念，鼓励自谋职业和自主创业。完善就业培训和服务体系。强化劳动执法，维护劳动者权益。

继续把“两个确保”放在重要位置，落实“三三制”筹资办法，依法强化社会保险金的征缴、扩面、清欠以及资金的管理，确保企业离退休人员基本养老金和国有企业下岗职工基本生活费按时足额发放。规范城市“低保”，合理确定社会保障标准，将长期亏损、停产、半停产困难企业符合条件的在岗职工全部纳入“低保”范围，切实做到动态管理下的应保尽保。进一步深化“心连心、双联系”帮扶活动，大力发展城乡社会救济和社会福利事业，切实安排好贫困地区群众的生产生活。

（十）强化安全生产监督管理，整顿和规范市场经济秩序

认真贯彻落实《安全生产法》，全力做好安全生产工作。突出煤矿安全管理这一重点，坚决落实市政府关于安全生产工作的四个政府令以及严防死守责任制，进一步加大稽查力度，严厉打击违法违规生产行为。对不符合安全生产条件和未通过省市验收的乡（镇）煤矿，一律予以关闭，并防止已关闭的小煤矿死灰复燃；对违法违规生产的小煤矿，不论是否发生事故，坚决予以关闭。认真落实各项安全生产责任制和事故责任追究制，搞好对危险化学品储运、非煤矿山、道路和水上交通、风景区、食品卫生、民爆器材和烟花爆竹等方面的安全管理，突出对影剧院、宾馆、学校、商场等公共聚集场所和重点部位的消防安全检查，加强对事故隐患和危险源的综合治理。切实做好森林防火工作。

继续深入开展社会各界评议政府部门活动。建立健全服务公开、承诺服务、限时办结、失职追究等相关制度，完善管理监督机制。严厉打击制售假冒伪劣商品特别是制售假劣食品、药品、医疗器械、农资等违法犯罪活动。加强生猪定点屠宰管理。加大矿业秩序整顿力度。整顿规范建筑市场。严厉查处“四乱”行为，进一步改善企业经营环境；对破坏经济秩序、严重影响企业经营环境、损害平顶山形象的重大案件，要依法从重从快查处。

四、加强社会主义精神文明和政治文明建设，大力繁荣各项社会事业

（一）继续实施“科教兴平”和可持续发展战略

进一步增强科技创新对经济发展的支撑作用。 以企业技术创新为主体，加强与大专院校和科研院所的合作，大力推进产学研结合。充分发挥大企业的技术中心作用，增强企业的技术创新能力。深化科研院所改革，完善内部经营机制。积极鼓励发展民营科研机构。进一步落实技术要素参与收益分配的政策，促进科研成果转化。继续加大科技投入，全面推行课题制和科研项目招投标制，引导更多的社会资金投向高新技术研究开发及产业化发展。大力扶持科技型中小企业，继续搞好农村科普工作。积极实施人才战略，依靠市场机制配置人才，造就一批管理型、科技型、特殊技能型人才，积极引进国内外高层次人才和优秀青年创业人才。

全面实施素质教育，大力发展教育事业。进一步落实“以县为主”的农村义务教育新体制，巩固“普九”成果，提高“两基”水平。增加教育投入，加强师资队伍建设，大力推进教育信息化，改善中小学办学条件。努力普及高中阶段教育，支持市一中争创国家级示范性高中，市一高、市二中争创省级示范性高中。优化高等教育结构，搞好平顶山工学院建设，加快师专升本科、质量工程学校升大专步伐。多渠道筹集教育资金，鼓励社会力量办学。大力发展职业教育、成人教育、社区教育，促进教育事业的全面发展。

实施文化精品战略，繁荣文学艺术、广播电视和新闻事业。加强文化产业建设，做好汝官窑遗址等文物古迹的保护和开发工作。依法加强文化市场管理，深入开展文化娱乐场所整顿。继续推进医药卫生体制改革，完善药品集中招标采购制度。优化卫生资源配置，健全社区医疗服务体系，大力加强农村医疗卫生工作。积极开展群众性体育运动，提高竞技体育水平，办好市第六届运动会。进一步推进广播电视、新闻改革，增强其实力和活力。继续做好民族宗教、对台、侨务、档案、史志、气象、残疾人、老龄等工作。加强国防教育和拥军优属工作，认真落实优抚安置政策，加快民兵预备役和人防工程建设，进一步做好创建全国双拥模范城工作。

以稳定低生育水平为目标，落实人口与计划生育目标管理责任制，认真做好国家在我市的计划生育综合改革试点工作，确保计生工作在全省的先进位次。大力推进殡葬改革。加强对资源的保护和合理利用，扩大国土资源有偿使用范围，规范完善土地市场和矿权市场。加大生态建设和环境保护力度，加快鲁山生态示范县的建设步伐。以防治大气污染为重点，全面推进“蓝天工程”。继续加强水污染治理力度，巩固白龟山水库综合治理成果。

（二）加强社会主义精神文明和政治文明建设

深入进行党的基本理论、基本路线、基本纲领和“三个代表”重要思想的宣传教育，贯彻落实《公民道德建设实施纲要》，广泛进行社会公德、职业道德、家庭美德和诚实守信教育。以创建文明城市、文明景区为载体，深入开展文明单位、文明行业、文明社区、文明村镇等群众性精神文明创建活动。继续推进“百县千乡”宣传文化工程，促进农村精神文明建设全面发展。

发展民主政治，建设社会主义政治文明。各级政府要依法向人大及其常委会报告工作，自觉接受监督，认真履行和落实有关决议、决定。加强与政协的协商与沟通，接受人民政协的民主监督。主动听取民主党派、工商联、无党派民主人士和各人民团体的意见和建议，加强与工会、共青团、妇联等人民团体的联系。扩大基层民主政治建设，加强社区管理与服务，进一步完善基层民主选举制度。全面推行行政执法责任制和责任追究制，加大执法监督力度。认真搞好“四五”普法工作，提高公民法律素质。

强化社会治安综合治理。继续深入开展“严打”整治斗争，严厉打击各种刑事犯罪和经济犯罪活动，维护社会治安秩序和市场经济秩序。加强治安防控体系建设，市县乡都要建立联防队伍，设立治安中心值班室和卡点，逐步建立“打防控”长效工作机制。大力加强信访和人民内部矛盾纠纷排查调处，积极防范、妥善处置群体性事件。

（三）进一步转变政府职能，切实改进工作作风

在大力推进改革开放和现代化建设的新形势下，加强政府自身建设特别是作风建设，十分重要。各级政府要进一步转变职能，理顺政府部门职能分工，提高政府管理水平，努力形成行为规范、运转协调、公正透明、廉洁高效的行政管理体制。继续办好市行政审批服务中心，简化审批程序，提高服务水平，全面推进服务型政府建设。

各级政府和全体工作人员要认真学习胡锦涛同志在西柏坡学习考察时的重要讲话，务必继续地保持谦虚谨慎、不骄不躁的作风，务必继续地保持艰苦奋斗的作风，想问题、作决策、办事情，始终要想到我们的宗旨是为人民谋利益。各级政府工作人员时时都要想到自己是人民的公仆，人民的温饱、人民的权益应该是我们的行使权力的中心点。是否符合广大人民群众的根本利益，是人民政府判断是非的根本原则和最高标准。各级政府及政府各部门必须做到：对于市委、市政府的重大决策，要不折不扣地贯彻落实，对于事关经济发展全局和人民切身利益的事情，要雷厉风行，坚决照办。以期形成说一句，算一句，句句算数；干一件，成一件，件件落实的局面。今年政府工作的任务十分繁重，我们一定要把重点工作和重大项目作为抓落实的关键，从市长到县（市、区）长和局长、主任，从县（市、区）长到乡（镇）长及各单位负责人，都要做到倾听社情民意在一线，真抓实干在一线，解决问题在一线。要形成抓落实的工作机制，按照进度，定期督促检查，公开检查结果，接受社会监督；要实行奖优罚劣，奖勤罚懒。

加强廉政建设，深入开展反腐败斗争。从教育、惩治、监督和制度建设入手，切实抓好领导干部廉洁自律、查处大案要案、纠正部门和行业不正之风工作。坚持标本兼治、综合治理的方针，深化各项改革，完善监督机制，从源头上预防和遏制腐败现象的发生。

各位代表，党的十六大为我们描绘了全面建设小康社会的宏伟蓝图，我市全面建设小康社会的目标任务和主要思路也都已明确。展望平顶山今后二十年的发展，我们既信心百倍，又深感责任重大。让我们在以胡锦涛同志为总书记的党中央领导下，高举邓小平理论伟大旗帜，全面贯彻“三个代表”重要思想，以十六大精神为动力，进一步坚定信心，克服困难，抢抓机遇，加快发展，紧跟全省全面建设小康社会的步伐，创造平顶山更加繁荣、美好的未来！
